# Endoscopic Hematoma Evacuation for Spontaneous Acute Subdural Hematoma: The Role of Preoperative Imaging

**DOI:** 10.7759/cureus.78710

**Published:** 2025-02-07

**Authors:** Takashi Iimori, Tatsuya Tanaka, Eiichi Suehiro, May Pyae Kyaw, Takashi Agari, Kazuaki Shimoji, Takashi Sugawara, Hiroshi Itokawa, Keisuke Onoda, Akira Matsuno

**Affiliations:** 1 Department of Neurosurgery, International University of Health and Welfare Narita Hospital, Narita, JPN

**Keywords:** acute subdural hematoma, computed tomography angiography (cta), endoscopic hematoma removal, ruptured cerebral aneurysm, spontaneous acute subdural hematoma

## Abstract

Spontaneous acute subdural hematoma (SDH) is a rare but potentially life-threatening condition. We present the case of a 73-year-old man with spontaneous acute SDH in which a computed tomography angiography (CTA) demonstrated contrast extravasation from a cortical artery. The patient was successfully treated with endoscopic hematoma evacuation via a small craniotomy, minimizing surgical invasiveness. This case highlights the value of CTA as an essential initial imaging modality for identifying the bleeding source and guiding treatment, particularly for endoscopic hematoma removal with a small craniotomy.

## Introduction

Spontaneous acute subdural hematoma (SDH) is a rare but potentially life-threatening condition, with reported mortality rates as high as 37.2% [1]. Unlike typical traumatic acute SDH, which is usually caused by head trauma, spontaneous acute SDH is caused by various etiologies, including rupture of cortical artery, rupture of aneurysm, vascular malformation, coagulopathy, neoplasm, intracranial hypotension, and vascular inflammatory diseases [2]. Therefore, preoperative imaging, including computed tomography angiography (CTA), is important for determining the treatment strategy for spontaneous acute SDH [3,4].

Here, we report a case of spontaneous acute SDH, initially suspected to be caused by a ruptured aneurysm based on preoperative CTA but found to have nonaneurysmal cortical arterial bleeding during surgery, successfully treated with endoscopic hematoma removal. This case highlights the CTA finding in spontaneous acute SDH that may serve as an early indicator of ongoing bleeding and a valuable marker for surgical planning, especially for endoscopic hematoma removal with a small craniotomy.

## Case presentation

A 73-year-old man presented with nausea and dull pain on the left side of his head, symptoms that had awakened him from sleep. His medical history was notable for diabetic nephropathy, requiring dialysis three times a week, cerebral lacunar infarction, congestive heart failure, and arteriosclerosis obliterans of the left lower limb. His regular medications included clopidogrel, cilostazol, carvedilol, vonoprazan, calcitriol, evocalcet, and nifedipine. The patient was independent in his daily activities. Both he and his family denied any recent head trauma.

**Figure 1:**
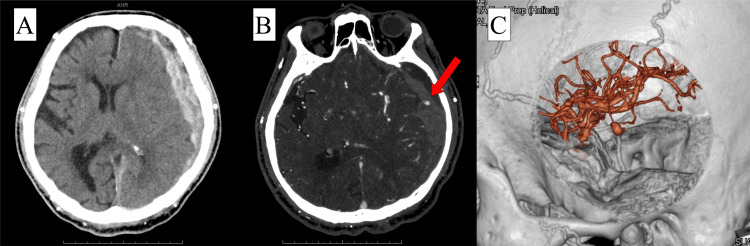
Preoperative images A: Computed tomography (CT) scan of the brain showing a 15-mm acute subdural hematoma on the left side. B and C: CT angiography with contrast showing contrast extravasation, raising concern for aneurysmal rupture (red arrow).

On examination, his vital signs were as follows: temperature 36.5°C, blood pressure 212/85 mmHg, pulse 86/minute and regular, respirations 17/minute, and pulse oximetry 99% on room air. He was alert and fully oriented, with a Glasgow Coma Scale (GCS) score of 15 (E4V5M6). Both pupils were 3 mm in diameter and reactive to light. Muscle strength was 5/5 in both upper and lower limbs bilaterally. A computed tomography (CT) scan of the head revealed a 15-mm acute SDH on the left side (Figure 1A). Initially, conservative treatment was selected due to the mild nature of his symptoms. However, while under observation in the emergency room prior to hospital admission, the patient's condition deteriorated. He became minimally responsive, withdrawing the left upper and lower extremities in response to stimuli, with no movement observed in the right upper and lower extremities. His GCS score dropped to 8 (E2V2M4). A repeat CT revealed an enlargement of the SDH to 20 mm with a 15-mm midline shift. The CTA performed at that time showed contrast extravasation into the subdural space on the surface of the temporal lobe, raising suspicion of a cortical artery aneurysm (Figures 1B, 1C). No bone fractures or other traumatic findings were observed.

**Figure 2:**
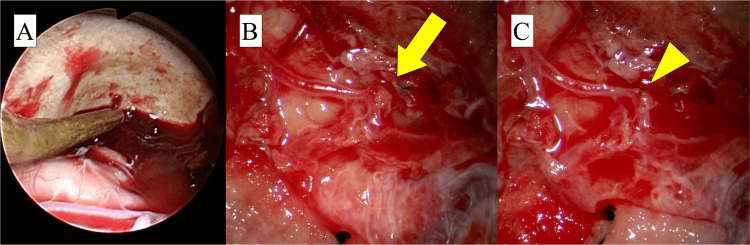
Intraoperative images A: Intraoperative photograph demonstrating the effective removal of the subdural hematoma using a suction tube with adequate maneuvering space under endoscopic visualization. B and C: Intraoperative images showing active arterial bleeding from a small perforation in a cortical artery (yellow arrow) with no evidence of an aneurysm and secure suturing of the perforation using 10-0 nylon under microscopic visualization (yellow arrowhead).

He was intubated and taken to the operating room for hematoma evacuation, identification of the source of bleeding, and hemostasis. Given his advanced age and pre-existing conditions, an endoscopic hematoma evacuation with a small craniotomy under general anesthesia was chosen to minimize surgical invasiveness. Considering the possibility of SDH associated with aneurysmal hemorrhage, the surgical team was prepared with a microscope and ready to promptly transition to a large craniotomy if necessary. A straight skin incision approximately 8 cm in length was made, followed by a 5 cm craniotomy directly above the point of contrast extravasation. After opening the dura and carefully evacuating the hematoma under endoscopic guidance (Figure 2A), active arterial bleeding was identified from a small perforation in a cortical branch of the middle cerebral artery at the center of the operative field (Figure 2B). Temporary hemostasis was achieved, and careful subarachnoid dissection revealed no evidence of an aneurysm. A 10-0 nylon suture was placed to close the arterial perforation under the microscope (Figure 2C). A drainage tube and an intracranial pressure (ICP) sensor were inserted into the subdural space. The dura mater was then closed, the bone flap was replaced with a titanium plate, and the scalp was closed.

**Figure 3:**
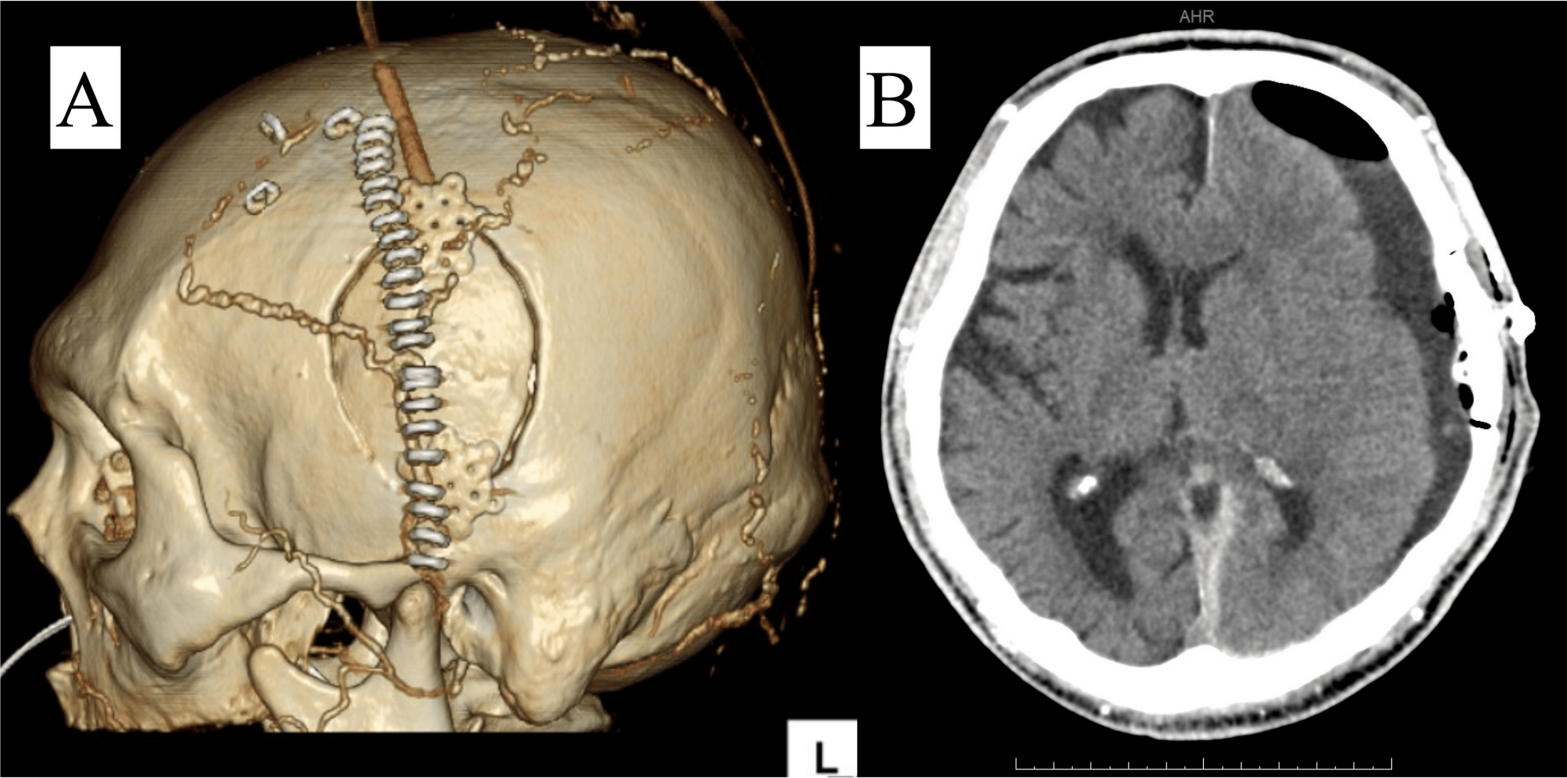
Postoperative images A and B: Postoperative images demonstrating complete removal of subdural hematoma with a small craniotomy.

Postoperative imaging demonstrated complete removal of SDH (Figures 3A, 3B). After surgery, the patient's GCS score was 14 (E4V4M6). The drainage tube was removed on postoperative day two, and the ICP sensor was removed on postoperative day five after confirming that the ICP was not elevated to a level requiring decompressive craniotomy. Right hemiparesis gradually improved, and he was subsequently transferred to a rehabilitation facility for further recovery.

## Discussion

Spontaneous acute SDH is rare compared to traumatic acute SDH [1]. However, given its mortality rate of up to 37.2%, early diagnosis and appropriate treatments are crucial [1]. CTA serves as a rapid and valuable initial imaging modality for identifying the cause and guiding treatment decisions due to its rapidity [3,4]. For neurologically stable patients, other modalities, including magnetic resonance imaging or digital subtraction angiography (DSA), should be considered for further evaluation [4].

In this case, preoperative findings on CTA suggested a possible rupture of a cortical artery aneurysm, but careful arachnoid dissection and thorough examination during surgery revealed no evidence of an aneurysm. A few similar cases have been reported, and Abecassis et al. first described this phenomenon as a "ghost aneurysm" [5,6]. The proposed mechanism suggests that a ghost aneurysm is a radiographic illusion caused by cortical artery extravasation into the subdural space, forming a partially spherical clot. Contrast accumulation within the clot during CTA creates a saccular appearance mimicking an aneurysm [5]. It is difficult to determine whether a spontaneous acute SDH is caused by a ruptured aneurysm or cortical artery bleeding solely on imaging studies. Even with DSA, it is difficult to make an accurate determination [6,7].

In cases of acute traumatic SDH, the presence of contrast extravasation is highly specific for hematoma expansion and can guide surgical intervention decisions [8]. Similarly, the presence of a "ghost aneurysm" should be recognized as a sign of ongoing bleeding and hematoma expansion. In this case, rapid hematoma growth and worsening consciousness suggest rebleeding, emphasizing the need for early intervention. Surgical planning should prioritize identifying the exact source of bleeding and addressing it with clipping, suturing, or coagulation, tailored to the nature of the lesion.

Large craniotomy has been the gold standard treatment for acute traumatic SDH [9]. However, it is often considered unsuitable for elderly patients due to their cognitive or systemic comorbidities [10]. Recently, several studies have described the validity of endoscopic surgery for acute traumatic SDH with a smaller craniotomy [11]. Endoscopic evacuation of hematoma with a smaller craniotomy is less invasive and may help prevent exacerbation of premorbid complications in appropriately selected cases [12]. In this case, given the patient's age, pre-existing conditions, and use of two antiplatelet medications, we prioritized minimizing surgical invasiveness. With no apparent history of trauma and no findings of brain contusion on imaging studies, we assessed that hematoma removal and proper treatment of the bleeding point would sufficiently reduce the risk of brain swelling and rebleeding. Although an aneurysm was considered a potential cause of the bleeding, CTA clearly identified the bleeding site. Thus, a small craniotomy directly over the bleeding point was planned to enable precise identification and treatment of it under a microscope, complemented by endoscopic hematoma removal as a minimally invasive approach. Additionally, an ICP sensor was implanted for early detection of brain swelling and rebleeding [11]. In cases of spontaneous acute SDH with a clearly identified bleeding site, endoscopic hematoma evacuation through a small craniotomy can be highly effective when accompanied by thorough preoperative evaluation and planning.

## Conclusions

We report a case of acute spontaneous SDH with preoperative CTA demonstrating contrast extravasation from a cortical artery. CTA serves as a useful initial imaging modality for patients with spontaneous acute SDH, providing crucial information to identify the cause and guide treatment decisions. Contrast extravasation should be recognized as an early indicator of ongoing bleeding and a potential marker for surgical planning, particularly for endoscopic hematoma removal with a small craniotomy.
